# Supplementary material for: Are carbon emissions trading and green financial instruments synergistic? -Comprehensive quantitative research based on content analysis
Source: PLoS One. 2024 Mar 7;19(3):e0298601. doi: 10.1371/journal.pone.0298601 (PMC10919601; doi:10.1371/journal.pone.0298601)
Supplement: S1 Data — (ZIP) [file pone.0298601.s001.zip › Data/地级市Z_F工作报告.zip!/║╙─╧/╨φ▓2╩╨/└┤╫╘╣1⁄2╓┌║┼í╛┬φ┐╦╩2╛▌═°í┐_2016.docx]

各位代表：

现在，我代表市人民政府，向大会报告工作，请予审议，并请各位政协委员和其他列席人士提出意见。

一、“十二五”主要成就及“十三五”目标任务

过去的五年极不平凡。我们在省委、省政府和市委的坚强领导下，深入贯彻落实党的十八大和十八届三中、四中、五中全会精神，扎实开展党的群众路线教育实践活动，致力打通“六路”，建设“五型许昌”，抢抓机遇，砥砺奋进，干成了一批打基础、利长远、惠民生的大事，“十二五”规划胜利完成，重大成就令人鼓舞！

（一）过去的五年，经济综合实力大幅跃升。全市生产总值达到2170.6亿元，人均生产总值突破5万元，均为2010年的1.7倍；规模以上工业增加值1294.2亿元，是2010年的1.8倍；一般公共预算收入138.5亿元，是2010年的2.4倍；累计完成固定资产投资7906亿元，是“十一五”时期的2.9倍；存款余额1733.1亿元，贷款余额1357亿元，分别是“十一五”末的2.1倍、2.4倍；规模以上工业增加值、生产总值、一般公共预算收入分别居全省第三、四、五位。经济综合实力稳居全省第一方阵！

（二）过去的五年，经济发展方式加快转变。工业经济增量提质，新增销售收入超百亿元企业6家，销售收入超百亿元民营企业占全省1/4强，装备制造业和战略性新兴产业成为工业发展的主导力量。产业集聚区主营业务收入占全市工业比重超过60%，成为经济最具活力增长极。第三产业增加值占生产总值比重达到33.2%，提高8.1个百分点，成为拉动增长、扩大就业的生力军。农业新型经营主体不断壮大，综合效益显著提升。高新技术企业和企业研发平台数量翻番，科技进步贡献率达58%，连续6次荣获全国科技进步先进市称号！

（三）过去的五年，城乡建设发展变化巨大。以人为核心的新型城镇化扎实推进，城镇化率年均提高1.7个百分点。中心城区首位度明显提升，建成区面积102.6平方公里，人口91.6万，城市功能不断完善，形象品质进一步提升。市城乡一体化示范区初具规模，县（市）城区和中心镇区快速发展，城乡环境综合整治和“美丽乡村”建设成效显著。成功创建国家节水型城市、全国创业先进城市，以优异成绩荣膺全国文明城市称号，获批创建国家生态文明先行示范区，跻身国家水生态文明城市、国家智慧城市、国家知识产权城市等建设试点行列。许昌知名度和美誉度大幅提升！

（四）过去的五年，基础支撑能力明显增强。石武客专建成通车，郑万、郑合高铁开工建设。中心城区至组团县（市）快速通道全部建成，城际公交全面开通。南水北调中线工程如期通水，三大水利项目全面实施。市区光纤和乡镇以上区域4G网络实现全覆盖。电力总装机突破260万千瓦。许都大剧院、博物馆、广电大厦等重大公共服务设施建成投用。许昌电气职业学院、河南农大分校建成招生，全民技能振兴工程和职教攻坚计划圆满完成。经济发展的支撑保障更加有力！

（五）过去的五年，改革开放取得重大进展。重点领域和关键环节改革取得突破，市级行政审批事项全省最少，农村、商事、食品药品、医药卫生等改革扎实推进。对外开放成就显著，累计出口84.2亿美元、实际利用外资25.8亿美元、实际利用省外资金1566亿元，分别是“十一五”时期的2.4倍、3.8倍和3.1倍。许昌出入境检验检疫局建成运营，许昌海关开关运行，大通关发展格局初步形成！

（六）过去的五年，人民群众得到更多实惠。积极顺应群众期盼，每年实施一批重点民生工程和民生实事，民生财政支出占比年均在70%以上。城乡居民人均可支配收入分别达到25225元、13355元，年均分别增长10.7%和13.2%。城镇新增就业46.5万人，农村劳动力转移就业新增52.3万人。企业退休人员养老金逐年提高，城乡低保标准分别提高55%、92%，农村五保对象集中供养、分散供养标准分别提高90%、133%。各级各类教育协调均衡发展，学前教育三年毛入学率由62%提高到88.5%。医疗、养老等社会保障体系覆盖城乡，医疗卫生机构床位数增长56%。建成保障性住房37952套。城市公共交通条件显著改善，被确定为全省首批“公交优先示范城市”创建市。新建农村公路2100公里，改造农村危房8090户，15.7万农村贫困人口稳定脱贫。改革发展成果更多惠及人民群众！

各位代表！“十二五”规划圆满收官，为“十三五”扬帆启航、开局起步奠定了坚实基础。这些成绩的取得，是省委、省政府和市委正确领导的结果，是市人大、市政协大力支持的结果，更是全市人民敢拼会赢的结果。在此，我代表市人民政府，向全市广大干部群众、驻许部队、武警官兵、公安民警致以崇高的敬意！向关心支持许昌发展的各界人士表示衷心的感谢！

“十二五”成绩来之不易，经验弥足珍贵，我们的主要体会是：必须始终坚持党的领导、强基固本，打造引领科学发展的坚强核心；必须始终坚持承天接地、科学谋划，坚定走具有许昌特色的发展路子；必须始终坚持问题导向、克难攻坚，保持稳中有进有为的良好态势；必须始终坚持以人为本、执政为民，顺应广大人民群众的愿景期盼。

各位代表！“十三五”时期是全面建成小康社会的决胜阶段，也是我市基本实现现代化建设大格局、争做中原崛起“领头雁”的关键时期，必须以滚石上山的勇气和精神，不断开拓发展新境界。

根据市委建议，市政府编制了“十三五”规划纲要（草案），已与纲要说明一起印发会议，提请审议。

“十三五”时期经济社会发展的指导思想是：高举中国特色社会主义伟大旗帜，全面贯彻党的十八大和十八届三中、四中、五中全会精神，以马克思列宁主义、毛泽东思想、邓小平理论、“三个代表”重要思想、科学发展观为指导，深入贯彻习近平总书记系列重要讲话精神，坚持“五位一体”总体布局和“四个全面”战略布局，坚持发展第一要务，坚持创新、协调、绿色、开放、共享的发展理念，坚持致力打通“六路”，加快建设“五型许昌”，推进四化同步科学发展，率先全面建成小康社会。

总体发展目标是：“两高一率先”。“两高”即主要经济指标增速高于全省全国平均水平，主要人均指标高于全省全国平均水平，“一率先”即率先全面建成小康社会。具体言之，就是经济发展实现新跨越，在提高发展平衡性、包容性、可持续性的基础上，力争2018年实现地区生产总值和城乡居民人均收入在2010年基础上翻一番；人民生活水平实现新提高，居民收入增长和经济发展同步，城乡居民收入进入全省第一方阵；新型城镇化建设实现新提升，常住人口城镇化率达到55%以上，中心城区人口达到120万人左右；“五型许昌”建设实现新突破，人民群众幸福感显著增强；治理体系和治理能力现代化迈出新步伐，重要领域和关键环节改革取得决定性成果，法治许昌建设全面推进。到2020年，四化同步发展水平走在全省前列，全市综合实力和综合竞争优势大幅提升，成为中原崛起河南振兴的“领头雁”之一！

围绕发展目标，必须认真落实“一极两区四基地”的发展定位：“一极”，即中原城市群重要增长极；“两区”，即全国生态文明先行示范区、全国创业创新示范区；“四基地”，即先进制造业基地、临空经济基地、现代物流基地、生态健康养生基地。必须加快构建“一带十区四组团”的发展布局：“一带”，即许港产业带；“十区”，即十个省级产业集聚区；“四组团”，即四个城市组团。必须大力实施创新驱动、开放带动、人才强市、许港融合四大发展战略。

实现发展目标，必须进一步明确重点工作任务，着力在五个方面取得重大突破。一是坚持创新发展，培育科学发展新动力；二是坚持协调发展，构建均衡融合新格局；三是坚持绿色发展，展现生态文明新形象；四是坚持开放发展，打造内陆开放新高地；五是坚持共享发展，创造幸福美好新生活。

各位代表!蓝图绘就未来，伟业催人奋进！我们坚信，经过五年不懈努力，我们一定能够续写更加辉煌的历史新篇章！

二、2015年工作回顾

过去一年，我们按照市委决策部署，主动适应经济发展新常态，统筹做好稳增长、调结构、促改革、惠民生、防风险各项工作，深入开展“三严三实”专题教育和“三查三保”活动，较好完成了市六届人大五次会议确定的目标任务。全市生产总值增长9%，一般公共预算收入增长10.6%，规模以上工业增加值增长9.1%，固定资产投资增长17.9%，社会消费品零售总额增长12.8%，存款余额增长13.1%，贷款余额增长16.4%，主要指标增速均高于全省平均水平。我们主要做了以下工作：

（一）积极作为稳增长，经济保持较快发展。把稳增长作为全局工作的突出任务。适时打出稳增长“组合拳”，制定实施了促进经济增长32条、财政支持稳增长29条、稳增长保态势20个重大专项等政策措施，经济运行总体平稳。把稳工业作为稳增长的首要任务，强化企业服务，着力化解企业财务风险，设立了8.44亿元的企业发展互助资金，为157家企业调贷45.3亿元。积极开展银企对接，着力化解融资难题，新增存贷比、余额存贷比分居全省第一、二位，新增新三板挂牌企业12家、区域性股权交易中心挂牌企业15家，资本市场直接融资96.4亿元。把稳定房地产市场作为稳增长的重要环节，坚持需求、供给双向发力，实行购房财政补贴政策等措施，房地产投资和销售面积分别增长10.1%、32.8%。把防范区域性系统性风险作为稳增长的必要保障，置换和新增政府债券40.5亿元，妥善处置非法集资，帮助银行化解不良贷款，金融生态环境持续优化。

（二）突出重点扩投资，发展后劲蓄积增强。把扩投资作为拉动经济增长的关键。组织实施“16718”投资促进计划，160个重点项目完成投资758亿元，带动固定资产投资1931亿元。坚持市级领导联系项目、联审联批、模拟审批等推进机制，新能源汽车产业园、1000兆瓦光伏发电等73个项目开工建设，许昌烟机易地技改、森源电气产业基地二期等33个项目竣工投产。加强政银合作，获得授信额度920亿元。推广政府和社会资本合作模式，推介PPP项目157个，33个入选省PPP项目库，争取省扶持资金5883万元，签约项目18个、总投资276亿元。

（三）分类施策调结构，质量效益明显提升。把优化经济结构作为动力转换的重要途径。加快工业转型升级，实施十大产业链发展计划，10个产业集群主营业务收入均超百亿元，装备制造业集群主营业务收入达到1609亿元，产业集聚区对工业增长的贡献率超过90%。战略性新兴产业主营业务收入增长20%以上，高技术产业增加值增长15.2%。实施“1242”服务业发展计划，全市服务业增加值增长12.2%。加快服务业“两区”建设，39个服务业项目完成投资90亿元。大力发展电子商务，许昌电子商务产业园、长葛空港电子商务产业园等开园运营，国内首家地市级阿里巴巴跨境电商产业带成功上线，全市涌现出5个淘宝村，应用电子商务企业3800家、增长52%，交易额达318亿元、增长44.5%。扎实推进农业“三大工程”，新增高标准粮田40万亩，建成千亩以上都市生态农业示范园区9个，粮食总产291.5万吨。强化创新驱动，设立中科院河南产业技术创新与育成中心许昌分中心、郑州大学许昌研究院，新增4家博士后科研工作站、12家高新技术企业、16家省创新型企业和创新型试点企业。建成市科技大市场。省级工程技术研究中心增量居全省第1位，新增专利申请量和授权量均居全省第3位。

（四）科学统筹促协调，城乡建设全面推进。把统筹城乡发展作为全面建成小康社会的根本要求。新一轮城市总体规划获得省政府批复。完善城市功能，实施“十大城建工程”，中心城区178个城建重点项目累计完成投资210.4亿元。加强城市管理，城市文明水平持续提升。坚持“一基本两牵动三保障”，科学推进新型城镇化，全市城镇化率达到47.57%。禹州市被列入国家深化县城基础设施投融资体制改革试点县，大周等4个乡镇被列入河南省首批重点建设示范镇。扎实建设新农村，新建沼气池1020口，新改造农村危房1605户，新建农村公路350公里。全面加强农村人居环境综合整治，新增省级生态乡镇5个、省级生态村13个，6个“美丽乡村”纳入省级试点，鄢陵县荣获省级生态县称号。

（五）着力兴水抓生态，绿色发展成效显著。把绿色发展作为竞争发展新优势。实施蓝天、碧水、乡村清洁工程和城市河流清洁行动计划，加强节能减排和生态建设，省定节能减排目标全面完成。强力推进大气污染防治，完成6个工业项目治理，注销黄标车16312辆，市区PM10、PM2.5明显下降。持续推进清潩河流域环境综合整治，五年水变清目标提前1年实现。倡导绿色交通，中心城区实行个人购买电动汽车财政补贴，建设电动汽车充电设施。实施循环发展引领计划，长葛成为国家循环经济示范县，大周再生金属循环产业集聚区、襄城循环经济产业集聚区进入省级行列。南水北调生态廊道建设率先完成，新建生态林20.4万亩。

紧紧抓住创建全国水生态文明城市的难得机遇，集中力量组织实施了中心城区河湖水系连通工程，三年任务两年完成，“五湖四海畔三川，两环一水润莲城”的水系格局已经形成，“河畅、湖清、水净、岸绿、景美”的新许昌呈现在世人面前。特别是有着500年历史的护城河重焕生机，“泛舟河上、环游古城”成为美好现实，许昌由一个严重缺水城市变成了“北方水城”，圆了几代许昌人的兴水之梦，许昌也因此被网民推选为全省“最美城市”！

（六）改革开放增动力，发展空间更加广阔。把改革开放作为关键一招，释放“制度红利”。重点领域改革扎实推进。市县两级政府机构、公务用车改革顺利完成。行政审批制度改革成效明显，市级行政审批事项压减至98项、办理时限缩减62.2%，市政府工作部门权责清单全面发布实施，精简压缩行政职权690项。公共资源交易管理改革扎实有效。商事制度改革加快推进，新增各类市场主体3.16万户、注册资本449亿元。率先在全省成立市级不动产登记局。产业集聚区与乡镇行政区域管理完成套合。医药卫生体制等改革顺利推进，获批建设国家中医药综合改革试验区。农村土地承包经营权确权登记颁证试点工作有序展开。深入推进开放招商，利用节会和重点区域定向招商成效显著，全年实际到位省外资金406.8亿元、增长8.8%。主动参与“一带一路”战略，与德国、东盟等国家和地区的有关组织签订合作协议，与4个国家的5个城市结为友好城市，许昌国际“朋友圈”越来越大，对外交流合作层次越来越高!

（七）顺应民意办实事，人民生活持续改善。把保障和改善民生作为政府一切工作的出发点和落脚点。十项重点民生工程、十件民生实事圆满完成。创业就业优惠政策全面落实，城镇新增就业9.6万人，农村劳动力转移就业新增9.5万人。加强社会保障，企业退休人员养老金人均月增长210元，城乡居民基础养老金月人均提高到84.8元，城乡低保月人均补助水平分别提高到不低于225元和115元；建成3个县级社会福利中心。每千名老人拥有养老床位提高到30.9张。推进教育均衡发展，突出解决中心城区大班额、入园难等问题，将许昌学院、许昌职业技术学院老校区置换为中小学，改扩建中小学15所，新建、改扩建幼儿园31所，市区优质初中教育资源覆盖率达到80%以上；年度农村义务教育“全面改薄”任务圆满完成。大力发展医疗卫生事业，城镇居民医保和新农合人均财政补助标准均提高到380元，人均基本公共卫生服务经费补助标准达到40元，市中心医院新院区开工建设，8个卫生重点项目快速推进，60个基层中小型卫生建设项目全部竣工。大力发展文化事业，创精品、扬正气，大型现代豫剧《燕振昌》作为重点剧目将在全省巡演。完善公共服务，建成第三期城市公共自行车系统，已投入公共自行车5000辆、设置站点240个，使用率居全国前列。启动市区农产品市场体系建设，中原国际农产品物流港一期建成运营，新建改建8家农贸市场、20个社区生鲜便利店，群众生活更加方便。保障性安居工程扎实推进，新建各类保障性住房16020套，基本建成8891套，3.1万人喜迁新居。实施精准扶贫，3.18万人成功脱贫。

一年来，我们自觉接受人大、政协和社会监督，认真办理人大代表建议和政协提案，支持民主党派、工商联和无党派人士参政议政。深入推进依法治市和服务型行政执法，着力打造“阳光政府”，政务公开范围更广，市长信箱、市长热线等社情民意渠道更宽，群众安全感和对政法部门的满意度分别居全省前2位。扎实开展“三严三实”专题教育，全面加强政府廉政建设，行政监察和审计监督作用进一步增强，行政效能持续提升，全市呈现出向好向快、向上向善、向富向强的良好局面！

与此同时，军民融合深入发展，外事、侨务、港澳、对台工作全面加强，妇女儿童、残疾人、慈善、红十字等事业健康发展，民族宗教、地方史志、人民防空、对口援疆、统计、体育、档案、气象、地震等工作都取得了新的成绩。

各位代表！我们也清醒认识到，经济社会发展还存在一些突出矛盾和问题：一是经济下行压力较大，投资、出口增长乏力，工业等部分行业困难加剧；二是转型升级任务艰巨，结构性体制性矛盾依然突出，现代服务业发育不足；三是城镇化水平低，城镇化率特别是户籍人口城镇化率低于全国平均水平；四是环境污染特别是空气质量问题依然突出，严重雾霾天气频发；五是风险隐患增多，潜在矛盾逐步暴露，要素约束依然突出；六是政府职能转变还不到位，一些干部不作为、不愿为、不会为问题依然存在，极少数干部以权谋私、贪污腐败。对此，我们一定要高度重视，认真解决。

三、2016年重点工作

今年是“十三五”规划开局之年。总的来看，今年还将继续面对复杂严峻的宏观经济形势，机遇与挑战并存，但机遇大于挑战。我们要坚定信心，切实打好供给侧结构性改革攻坚战，确保全面完成年度各项目标任务。

今年政府工作的总体要求是：全面贯彻党的十八大，十八届三中、四中、五中全会和中央、省委、市委经济工作会议精神，以“五位一体”总体布局和“四个全面”战略布局为统领，牢固树立和贯彻落实创新、协调、绿色、开放、共享发展理念，坚持改革开放，坚持稳中求进工作总基调，坚持稳增长、调结构、惠民生、防风险，深入推进创新驱动，加强供给侧结构性改革，适应新常态、抢抓新机遇、培育新动力、打造新优势，致力打通“六路”，建设“五型许昌”，确保实现“十三五”开好局、起好步，为率先全面建成小康社会打下坚实基础。

今年经济社会发展的主要预期目标是：生产总值增长8%以上，一般公共预算收入增长8%以上，规模以上工业增加值增长9%以上，服务业增加值增长9.5%以上，全社会固定资产投资增长15%，社会消费品零售总额增长12%，进出口总值增长7%，实际利用外商直接投资和省外资金均增长7%，城镇新增就业6.6万人以上，城乡居民收入增长速度与经济增长同步，居民消费价格涨幅控制在3%左右；节能减排完成省定任务。

贯彻总体要求，实现预期目标，做好今年政府工作，必须切实做到“六个更加注重”。一是更加注重创新驱动发展。坚持把创新驱动作为引领发展的第一动力，大力推动大众创业、万众创新，加快构建产业创新体系，加强科技创新平台建设，培育发展新动力，提高发展质量和效益。二是更加注重开放带动发展。坚持把扩大开放作为繁荣发展的必由之路，主动参与“一带一路”，加快许港产业带建设，积极承接产业转移，持续完善开放平台，不断开拓发展空间。三是更加注重厚植发展优势。坚持发挥有效投资对稳增长调结构的关键作用，抓牢项目建设抓手，扩大投资规模、优化投资结构、提升投资效益，增创发展优势，持续提高发展竞争力。四是更加注重供给侧结构性改革。坚持把供给侧结构性改革作为推动发展的重大举措，以改革开放创新促进要素优化配置，打好去产能、去库存、去杠杆、降成本、补短板“五大攻坚战”，加快形成引领经济发展新常态的体制机制和发展方式。五是更加注重保障和改善民生。坚持把改善民生作为科学发展的出发点和落脚点，大力实施十项重点民生工程和十件民生实事，增加公共服务供给，完善社会保障，以民生改善促进发展，发展成果由人民共享。六是更加注重防范和化解风险。坚持把防控风险作为经济平稳运行的基础，增强忧患意识和责任意识，创新社会治理，坚决防止各类风险相互传导、相互叠加，坚决守住不发生区域性系统性风险的底线，保持社会大局和谐稳定。

（一）扎实推进供给侧结构性改革，推动工业转型升级。坚持优化存量、引导增量、主动减量，抓好“三去一降一补”，为经济发展增添新的动力。

做好“加减乘除”优结构。做好加法，重点抓好新能源汽车、工业机器人、生物医药三大战略性新兴产业，加快新能源汽车产业园、工业机器人及智能制造产业园、市中医药产业园、禹州市医药健康产业园建设，大力扶持科技型中小企业，培育高新技术企业、创新型企业，加快形成新的经济增长点。做好减法，用好国家去产能政策，切实化解煤炭、水泥等过剩产能，分类引导企业加快转型发展，鼓励支持困难企业兼并重组，有序实现市场出清。做好乘法，借助实施“互联网+”行动计划、“中国制造2025”许昌行动纲要，用好科技大市场、科技企业孵化器、众创空间、科技银行等载体平台，对共性关键技术重点攻关，加快电力装备首席产业链发展，推动六大优势产业链转型升级。做好除法，扩大分子、缩小分母，提高劳动生产率、资本回报率；同时，加大简政放权力度，严格落实减负政策，严厉查处“四乱”行为，切实减轻企业负担。

加快载体建设转方式。深入实施产业集聚区提升计划，加快效益型、品牌型和创新型产业集聚区建设。实施集群培育工程、提质创新工程和配套提升工程，推动魏都、襄城循环经济、大周再生金属循环3个产业集聚区晋升一星级，禹州市、长葛市产业集聚区晋升三星级。力争2016年产业集聚区规模以上工业主营业务收入突破4000亿元、固定资产投资达到1300亿元、超百亿元特色产业集群达到12个。

做好服务企业强主体。实行领导分包联系企业制度，加强产销、银企、用工、产学研对接，协调保障要素，加大对实体经济特别是经营困难企业的帮扶力度。实施大企业集团培育计划和企业家队伍培养“十百千”行动计划，引导支持企业争做行业领军企业。加快企业上市步伐，年内新增主板上市企业1—2家、“新三板”挂牌企业10家以上。加快培育发展中小微企业，推动形成大企业“顶天立地”、中小微企业“铺天盖地”、“专精特新”企业“占领高地”的良好局面。

（二）加快服务业提升发展，打造经济增长新引擎。把服务业作为产业结构调整的重点和突破口，大力实施“1242”服务业发展计划，推动服务业发展提速、比重提高、结构提升。

大力发展信息服务业。以智慧城市建设为抓手，加强公共信息服务平台建设，推进大数据发展应用。加快市、县两级电子商务产业园发展，争取我市纳入中国（郑州）跨境电子商务综合试验区。加强与阿里巴巴、云书网、河南广电喜买网在跨境电商、人才培养、农村电商等领域的合作，做强鲜易网、日日鲜、冷链马甲等品牌，加快鄢陵县国家级电子商务进农村示范县建设，打造在全国有影响力的电商示范基地。

加快服务业“两区”建设。全面完成服务业“两区”规划调整。抓好49个千万元以上产业项目，完成年度投资85.7亿元。突出商务中心区生产性服务功能，加快芙蓉湖周边开发，推动总部经济、金融商贸、文化创意等业态集聚，打造芙蓉湖楼宇经济圈。增强特色商业区生活性服务功能，建设一批业态配比合理、功能复合多元的城市综合体，推动特色商业区全面提升。

培育壮大现代服务业。积极对接郑州航空港经济综合实验区，加快发展现代物流业，推动中原国际农产品物流港二期、许昌粮食物流园、中棉集团河南物流园等专业园区加快建设。强化投入，注重管理，持续打造文化旅游业，加快构建以市区三国文化和生态水系景区、鄢陵旅游度假区、禹州古镇体验景区为支撑的“三点一线”旅游体系。着力发展现代金融业，支持在许各商业银行加快发展，主动引入外埠金融机构，鼓励设立民营银行，支持有条件的企业设立金融租赁、财务公司等非银行金融机构。积极发展健康养老服务业，重点推进河南花溪科学养老中心、河南建业生态养老示范项目、中国科学养老河南鄢陵示范基地、河南龙耀健康城等综合性、基地型养老项目，努力形成全省重要的生态健康养生基地。

改造提升传统服务业。定期研判房地产市场变化，实行动态调控，促进房地产业健康发展。大力发展住房租赁和二手房市场，畅通商品房供给保障性住房通道，提高棚改货币化安置率，鼓励房地产业兼并重组和发展跨界地产。改造提升商贸流通业，重点推进东城区商圈建设，推进市区农产品市场改造提升。大力发展连锁经营、折扣店、工厂店等新型业态，支持传统商贸企业建设网上商城、拓展农村经营网络。

顺应生产生活需要，大力发展社区服务、商务服务、科技服务、专业性生产服务、农村服务等服务业。组织实施好万达商业广场、深国投家乐福、万盛国际广场等一批重大服务业建设项目。

（三）扎实做好“三农”工作，夯实经济发展基础。把解决好“三农”问题放在全部工作的重中之重，厚植农业农村发展优势，推进农业现代化，提高农业综合效益。

稳定提高粮食产能。落实藏粮于地、藏粮于技战略，加快实施高标准粮田“百千万”工程，新建高标准粮田30万亩，发展高效节水灌溉面积21.3万亩。大力推广农业先进适用技术，加大农业技术服务指导，深入开展粮食绿色增产示范创建工作，调整粮食种植结构，提高土地综合产出效益，建设优良品种繁育基地30万亩，建立农作物病虫害防控示范区。

加快发展现代农业。推动农业产业化经营，加快6个省级农业产业化集群建设；加快现代畜牧业集群发展，推广种养结合模式，着力提高畜牧业生产的质量效益和竞争力；大力推进“互联网+现代农业”发展，鼓励一二三产业融合发展示范县建设，以农产品加工、物流业为重点，规划建设一批乡村特色经济园区和特色产业集群。大力发展都市生态农业，科学编制发展规划，着力培育农业观光、体验农场、休闲农庄等都市生态农业新业态。大力培育种养大户、家庭农场、农民专业合作社等新型农业经营主体，新培育农民合作社500家、家庭农场200家。培养更多的新型职业农民。让农民成为体面的职业！让农业成为有奔头的产业！

提高新农村建设水平。加快基础设施和公共服务设施向农村延伸，新建农村公路480公里，新改造农村危房800户，新增及提升农村安全饮水人口10万人。持续开展农村人居环境综合整治，实施“三保三化”，建立垃圾、污水处理长效机制，加强改善农村人居环境达标村、示范村创建工作，抓好省级“美丽乡村”试点项目建设。让农村成为安居乐业的美丽家园！

（四）积极扩大有效投资，增强经济发展后劲。发挥好有效投资对稳增长调结构的关键作用，优化投资结构，注重定向精准，弥补发展“短板”，持续拉动经济增长。

大力实施“1782”投资促进计划。在先进制造业、高成长性服务业、社会事业、农林水利、交通能源、新型城镇化等重点领域实施170个重点项目，完成年度投资800亿元，带动全市固定资产投资2000亿元以上。

加快重大基础设施建设。提升现代交通优势，扎实推进禹亳铁路和郑万高铁、郑合高铁许昌段等重大铁路项目，加快许昌至机场快速通道和国道230线、国道107线许昌境改建工程，争取已获得“路条”的7条干线公路开工建设。强化能源保障能力，抓好许昌城乡电网提升工程、500千伏许昌西输变电工程项目，加快禹州市山地林光互补光伏发电、襄城县紫云风电场等项目建设。持续强化水利支撑，突出抓好引黄入长济许调蓄工程等重点水利项目，做好禹州沙陀湖调蓄工程前期工作，全面完成50万亩高效节水灌溉工程。同时，加强重大项目谋划储备，不断厚植发展优势。

继续拓展融资渠道。用好国家扩大地方政府发债规模政策，争取第三期18亿元城投债和12亿元中期票据早日获批。充分发挥市级投融资平台的桥梁作用，探索建立城建重点项目融资建设新模式，积极争取社会资本和银行贷款支持。大力推广PPP模式，创新项目收益和分配机制，补充完善PPP项目储备库，争取更多项目进入省项目库并签约实施。

（五）统筹规划建设管理，推动城市内涵式发展。顺应城市发展规律，坚持“五大统筹”，做好规划、建设、管理“三篇文章”，致力建设生态宜居、富有活力、彰显特色的现代化城市。

做大做强中心城区。突出规划引领，严格依法制定、执行规划，增强规划前瞻性、严肃性、连续性；认真落实新一轮城市总体规划，完善各类专项规划，留住城市特有的地域环境、文化特色、建筑风格等“基因”，塑造城市特色风貌。坚持基础先行，加快六大片区、十大工程和199个城建重点项目建设，完成年度投资229亿元。推进智慧城市、地下综合管廊、海绵城市试点建设。坚持“一基本两牵动三保障”，加快户籍制度改革和居住证制度“双落地”，促进在城镇稳定就业和生活的农民有序实现市民化，提高户籍人口城镇化率。

彰显城市个性特色。着力提升中心城区发展内涵。以“绿”为美，构建公共绿地系统，打造“两环三带四海二十园”城市林带及城郊森林生态体系，让森林走进城市，让城市拥抱森林。以“水”为韵，持续做好水文章，实施护城河环通二期等20个水生态文明类项目，加快推进北护城河和西湖公园提升工程，启动小洪河城区段治理工程，实现河湖水系连通工程完美收官；以曹魏古城、鹿鸣湖、秋湖湿地开发为重点，统筹推进五湖三河两岸周边综合开发，实现生态效益、社会效益和经济效益最大化。以“文”为魂，传承历史文脉，突出曹魏文化，统筹旧城改造与文化塑造，提升城市文化内涵，塑造城市文化形象。以“人”为本，加强城市精细化、规范化、数字化、法治化管理，创建国家公交都市，巩固提升全国文明城市创建成果。我们要共同珍惜文明城市这块金字招牌，人人共建，人人共享，让许昌真正成为一座既有历史记忆、文化传承，又有时代气息、充满魅力的宜居宜业之城，成为中原大地上一颗璀璨耀眼的明珠！

加快城乡一体化示范区建设。按照“四区一基地”的发展定位，加快许继智能电网二期、祺景LED照明生产基地、开普国家电工电子产品检测平台等重点项目建设，以整机带集群，规划发展电机产业，着力把中原电气谷打造成国内一流、国际先进的电力装备研发监测中心、制造中心、系统集成中心和重要电力装备出口基地，尽快形成千亿级产业集群。加快商务中心区建设，支持森源、瑞贝卡、众品、黄河、腾飞集团等重点企业建设总部基地，规划建设数据中心，做大做强电商产业园，尽快形成总部经济基地和新兴业态基地。按照城乡统筹、产业融合、功能复合的要求，把城乡一体化示范区建成率先实现现代化的样板区和产城融合发展的示范区。

加快县（市）城区发展。科学编制县城和重点镇规划，推进县（市）城区开发建设。实施提升县级城市管理水平三年行动计划，扎实推进县级智慧城管建设，年底实现全市数字城管系统全覆盖。抓好禹州市国家新型城镇化综合试点工作，支持长葛市开展第三批综合试点申报工作。加快中心镇区建设，以神垕、大周等4个省首批重点示范镇为重点，建设一批产业特色鲜明、生态环境优美的特色小镇。

（六）深化改革开放创新，激发内在动力活力。聚焦重点领域和关键环节，强化改革推动、创新驱动、开放带动。

着力重点领域改革。深化财税体制改革，推进营改增扩围、消费税和资源税改革，加快市级财政性涉企资金基金化改革。深化商事制度改革，进一步放宽市场准入。深化投融资体制改革，探索建立企业投资项目负面清单制度，制定禁止类和限制类清单，积极争取国家、省级各项基金化改革资金的参股支持。深化金融体制改革，推进市农商行组建工作，争取县级农信联社全部达到组建农商行标准，加快推进资产证券化。深化农村改革，完成农村土地承包经营权确权登记颁证工作，健全土地流转服务体系和仲裁体系，提高土地流转规范化水平。抓好气象现代化试点市建设。加快企事业单位改革改制，统筹做好价格、教育、科技、文化、城管、公共资源交易等领域改革。

推进创新驱动发展。抓好国家创新型试点城市、知识产权示范城市、全国质量强市示范城市创建和国家级高新区申建工作，争取进入全省国家自主创新示范区。积极推进科技重大专项，实施省级以上科技项目30个、省级科技重大专项3—5个。加快企业研发平台建设，新建省级工程技术研究中心3—5个、市级工程技术研究中心15—20个，新培育一批省级科技企业孵化器。积极组建产业技术创新战略联盟，再争取与2—3所省外知名高校和科研院所签订战略合作协议。实施人才强市战略，加大人才培养和引进力度。深入推动大众创业、万众创新，引导更多社会力量参与众创空间建设。

提升开放招商水平。积极参与“一带一路”建设，支持有条件的企业“走出去”，鼓励优势企业开展国际产能、装备制造合作和跨境并购，争取建设中德（许昌）工业园。实施“5651”对外开放和招商引资行动计划，谋划推介500个重点招商项目，推动60个已签约5亿元以上项目落地，全年实际到位市外资金510亿元。创新招商方式，加快“四个转变”，推行“二分之一”工作法，办好三国文化旅游周、花博会和药交会等招商活动，每季度在发达地区举办一次专题推介对接活动，加强对签约项目跟踪问效。加快大通关开放平台建设，提升出入境检验检疫水平和海关通关效率，启动申建保税物流中心。全面推进跨区域通关便利化，建立“一站式”服务体系。稳定发制品等传统外贸产品出口规模，扩大机电产品、特色农产品、鲜切花等出口规模，提升外贸出口质量效益。

（七）持续改善生态环境，着力建设美丽许昌。全面推进国家生态文明先行示范区建设，深入实施蓝天、碧水、乡村清洁工程，为人民群众提供更多绿色空间。

实施更加有效的环境治理。实施蓝天工程，严格落实“五控”措施，突出抓好能源结构调整，实施燃煤电厂超低排放改造，加强城市扬尘污染防控、机动车污染防治，持续改善空气质量，为人民群众留住更多“许昌蓝”！实施碧水工程，建立河湖水系管护长效机制，确保清水长流。实施乡村清洁工程，加强农村生活污水、生活垃圾、畜禽养殖场等环境综合治理，着力打造清洁家园、清洁水源、清洁田园。

开展更加积极的生态建设。实行最严格水资源管理制度，确保饮用水源地水质达标和水环境质量安全。推动鄢陵县创建国家级生态县，全市完成2个省级生态乡镇、8个省级生态村创建任务。抓好生态防护林带、大型生态林地、都市生态林业产业园、南水北调中线生态走廊建设和山区生态修复，完成林业生态建设16.8万亩、矿区生态修复2000亩，筑牢生态安全屏障！

实行更加严格的环境管理。坚决杜绝“两高一资”、产能过剩行业和低水平重复建设项目，从源头上防止环境污染和生态破坏行为。实施节能减排降碳行动计划，在工业、建筑、交通、能源、公共机构等五大重点领域推广节能技术和装备，完成省定减排任务。全面实施排污许可、排污权有偿使用制度，完善环评审批与环境容量挂钩和差别化的环评审批制度，强化重点污染物总量预算管理。加大环境执法监察力度，严厉打击环境违法行为。我们要共同保护好我们的美丽家园，谁敢破坏我们的环境，就让他付出应有的代价！

（八）切实保障和改善民生，增进人民群众福祉。坚持问题导向，顺应群众需求，倾力办成一批民生工程和民生实事，让群众有更多现实获得感。

加快扶贫攻坚步伐。坚持精准识贫、精准扶贫、精准脱贫，按照“五个一批”要求，通过“转、扶、搬、保、救”途径，完成80个村、5.25万贫困人口脱贫任务，确保脱贫攻坚首战必胜！

稳定扩大就业创业。深入创建国家级创业型城市，继续实施高校毕业生就业促进和创业引领计划，高校应届毕业生综合就业率达到90%以上。加大就业援助力度，实现城镇新增就业6.6万人，新增农村劳动力转移就业6.3万人。深入实施全民技能振兴工程，开展各类职业技能培训15万人次。让群众创业有“助”、就业有“路”！

优先发展教育事业。深入推进全面改善义务教育薄弱学校基本办学条件和扩充城镇义务教育资源五年规划，完成农村学校“全面改薄”199个项目建设任务，实施中心城区中小学、幼儿园提速攻坚计划，建设中小学15所、幼儿园16所，扩充学位2.6万个。加快构建现代职业教育体系，规划建设职教园区，启动许昌工商管理学校扩建、许昌科技学校迁建工作。支持高校转型发展，推进产学研合作。鼓励民办教育全面健康发展。完善助学体系，推动中等职业教育免除学杂费，率先免除建档立卡家庭经济困难普通高中学生学杂费。让每个孩子都能接受公平有质量的教育！

提升人民健康水平。深化县级公立医院综合改革，支持民办医疗机构健康发展，推进基本公共服务均等化。抓好市中心医院新院区等9个卫生重点项目，争取市立医院一期工程上半年建成启用；改造提升53个基层医疗卫生机构，加快15个城市社区卫生服务中心建设。提升国家卫生城市创建成果。落实全面两孩政策，完善计划生育服务管理。努力打造健康许昌！

加强公共文化体育服务。健全公共文化、体育服务体系，加快职工之家、三国文化演艺中心建设，力争市科技馆建成投用，启动市文化艺术中心、老干部活动中心、全民健身活动中心、体育公园建设，着手规划市体育中心。大力实施文艺精品、文化惠民、体育健身等工程，加强农村、社区文化中心建设，丰富基层群众文体生活。让老百姓的生活更加多姿多彩！

完善社会保障体系。开展全民参保登记，推动社会保险全覆盖，深入实施机关事业单位养老保险制度，继续提高企业退休人员基本养老金，城乡低保财政补差水平月人均分别提高15元和12元，农村五保对象年集中供养、分散供养标准分别提高到人均4000元和3000元，落实好城乡医保制度整合工作。下大力气做好特殊困难群体救助帮扶工作，确保困难群体应保尽保、受助及时，切实兜牢民生保障底线。促进残疾人、妇女儿童、红十字、老龄、慈善等事业发展，健全农村“三留守”人员关爱服务体系。切实消除弱势群体后顾之忧！

各位代表！人民群众对美好生活的向往，就是我们的奋斗目标。今年，市政府在组织实施好各项民生工程的同时，重点办好十件民生实事。一是加强中心城区河湖水系建设管护，确保清水长流；二是解决中心城区入学难大班额问题，为群众提供更多优质教育资源；三是完善中心城区社区卫生服务，打造“15分钟健康服务圈”；四是实施中心城区养老服务网络工程，为老年人营造健康舒心美好的生活环境；五是实施中心城区“明厨亮灶”视频工程，保障人民群众舌尖上的安全；六是完善市区农产品市场体系，方便群众生活需求；七是改善困难群体住房条件，解决“千户万人”住房困难；八是建设许昌市电梯应急处置中心，保障居民乘梯安全；九是实施蓝天工程，持续改善空气质量；十是发展绿色交通，倡导和推动绿色出行。对于这十件民生实事，我们要一抓到底，件件落实，坚决兑现对人民群众的庄严承诺，让人民群众在件件实事中感受到许昌的变化，不断增强作为一个许昌人的幸福感和自豪感，让许昌成为一个更加令人向往的城市！

（九）有效防范和化解风险，促进社会和谐稳定。坚持底线思维，管控好各种潜在风险，最大限度维护群众利益、维护经济发展、维护社会稳定。

积极防范化解金融风险。加强宣传教育，让人民群众远离非法集资，落实属地管理责任和部门监管责任，加大处置和打击非法集资力度。加强政银企协作，采取综合举措，充分发挥企业发展互助资金作用，着力防范和化解企业财务风险。加强政府债务管控，优化债务结构，有效防范化解财政金融风险。帮助银行化解不良贷款，严厉打击逃废债行为，优化金融生态环境，坚决守住不发生区域性系统性金融风险底线！

切实加强安全监管。牢固树立安全发展理念，突出重点，落细落小，深入排查整改安全隐患，坚决遏制重特大安全事故发生。落实“四个最严”要求，强化日常检查和监督抽检，加强基层监管能力建设，严厉打击食品药品违法活动，坚决杜绝食品药品安全事故发生！

不断创新社会治理。着眼“双安”、强化“双基”、推进“双治”，提高社会治理水平。加强矛盾纠纷排查化解，注重依法化解社会矛盾。完善立体化社会治安防控体系，强化流动人口和特殊人群服务管理，加强网络舆论引导，依法打击违法犯罪活动，进一步增强人民群众的安全感！

同时，加强国防教育和国防后备力量建设，深入开展双拥共建，巩固军政军民团结，推动军民融合深度发展。支持工、青、妇等人民团体更好发挥桥梁纽带作用。促进民族团结进步、宗教关系和谐。做好外事、侨务、港澳、对台工作。加强统计、地方史志、档案、社科研究、地震、对口援疆等工作。

四、建设廉洁高效政府

打铁还需自身硬。我们要以踏石留印、抓铁有痕的劲头，统筹推进法治政府、创新政府、廉洁政府、高效政府和服务型政府建设，持续提升政府治理能力和水平。

（一）加快转变政府职能。坚持法定职责必须为、法无授权不可为，减审批、重服务、强监管。深入推进简政放权，全面落实权责清单，建立责任清单动态调整机制，适时调整行政权力目录。切实改进政府服务，推进行政许可和行政处罚网上“双公示”，设立网上“中介超市”，建设“市民之家”，推广“互联网+政务服务”，让数据多跑路、让群众少跑腿。加强事中事后监管，推行“两随机、一公开”检查，砍掉繁文缛节，切断利益输送，让群众办事更便捷、创业更顺畅！

（二）深化法治政府建设。强化法治思维，推进政府运行机制法治化，依照法定权限和法定程序行权履职。建立完善政府立法机制，认真做好地方立法工作。健全依法决策机制，认真履行重大决策法定程序。深化行政执法体制改革，全面落实行政执法责任制，加强行政执法监督，促进严格规范公正文明执法。启动“七五”普法。加强行政复议和行政应诉工作，让行政行为接受司法“体检”。依法接受人大及其常委会法律监督和工作监督，主动接受政协民主监督，自觉接受社会、司法和舆论监督，让行政权力始终在法治轨道和阳光下运行！

（三）强化政府执行能力。坚持主动作为，积极履职尽责，狠抓贯彻落实，真正做到勤政为民。建立完善工作落实的责任机制、激励机制，创新公共管理，着力营造敢于担当、善于作为的干事创业氛围。深入开展懒政怠政为官不为问责年活动，加强监督执纪问责，对“门好进、脸好看、事不办”现象，依法依纪严肃问责，下决心打通政策落实中的“中梗阻”和“最先最后一公里”！

（四）加强廉洁政府建设。践行“三严三实”，深入开展“两学一做”学习教育。从严落实党风廉政建设主体责任和“一岗双责”，切实扎紧制度笼子。加强廉政教育，突出家风建设，树立高尚职业道德和精神追求，做到干部清正、政府清廉、政治清明。强化权力运行制约监督，推动行政监察和审计监督全覆盖。紧盯干部作风突出问题，加大对群众身边“四风”和腐败问题的查处力度，始终保持反腐败高压态势！

各位代表！新的一年开启新的征程，新的征程承载新的梦想；许昌“十三五”新的发展蓝图，鼓舞我们砥砺奋进；全市人民对美好生活的新期盼，鞭策我们勇往直前。让我们在省委、省政府和市委的坚强领导下，凝神聚力，拼搏奋进，克难攻坚，务实重干，努力实现“十三五”良好开局，为建设“五型许昌”、率先全面建成小康社会而努力奋斗！
